# Socioeconomic effects on breast cancer survival: proportion attributable to stage and morphology

**DOI:** 10.1038/sj.bjc.6601339

**Published:** 2003-10-28

**Authors:** F Kaffashian, S Godward, T Davies, L Solomon, J McCann, S W Duffy

**Affiliations:** 1 Cancer Intelligence Unit, Strangeways Research Laboratory, Cambridge, UK; 2 Queen Mary College and Cancer Research UK, London, UK

**Keywords:** breast cancer, survival, socioeconomic status, stage, morphology

## Abstract

Breast cancer patients of lower socioeconomic status tend to have poorer survival. Among 10 865 cases of breast cancer from the East Anglian Cancer Registry diagnosed between 1982 and 1993, we estimated the extent to which the differences in survival by socioeconomic status, measured by both occupational and area-based methods, can be explained by differences between socioeconomic groups in stage and morphological type of tumour. In univariate survival analyses, lower social class (manual occupation) was associated with a relative hazard of 1.32 (95% CI 1.12–1.55) for death from breast cancer as underlying cause. Women resident in the most deprived area had a relative hazard of 1.21 (0.95–1.54) for death from breast cancer as underlying cause. Stage of disease accounted for 28% of the effect of social class on survival but for none of the effect of deprivation category. Morphological type accounted for 3% of the effect of social class and none of the effect of deprivation category. Thus, stage at presentation explains some but not all of the socioeconomic differences in breast cancer survival. Future research on histological grade and socioeconomic status is indicated.

Ethnic and socioeconomic effects on breast cancer survival are a continuing issue in breast cancer research (Meng *et al*, 1997; Pollock and Vickers, 1997; Thomson *et al*, 2001; English *et al*, 2002; Newman *et al*, 2002). Although affluent women have a higher incidence of breast cancer than socially deprived, several studies using individual and area-based measures have shown that deprived women with breast cancer from low socioeconomic groups have poorer survival from disease (Carnon *et al*, 1994; Kogevinas and Porta, 1997; Meng *et al*, 1997; Pollock and Vickers, 1997; Thomson *et al*, 2001). Possible explanations of the finding include later diagnosis, and consequently a more advanced stage of tumour in poorer women, and differences in care and treatment of the cancer among women from deprived and affluent areas (Twelves *et al*, 1998). Previous studies suggest that neither explanation fully accounts for the phenomenon (Carnon *et al*, 1994; Macleod *et al*, 2000b; Thomson *et al*, 2001).

If the poorer prognosis of low-status women is mainly explicable by a higher stage of tumour at presentation, then we should intervene in the area of early detection. If a substantial and significant association between poorer prognosis and low socioeconomic status persists after adjusting for stage of tumour, then this would suggest that we should intervene in the area of treatment. If tumours in women with low socioeconomic status appear to be different *ab initio* in terms of aggressive potential, as measured by morphological/histological type, then this would point to more effective targeting of therapy to the tumour, and also suggests that such women may be suitable candidates for chemoprevention studies. In this study, we investigate the consequences of adjustment for stage and morphology in survival in 10 865 breast cancer cases in East Anglia.

## MATERIALS AND METHODS

In total, 10 865 breast cancer cases were included in our study drawn from the Eastern Anglian Cancer Registry, and diagnosed between 1982 and 1993 in four hospitals of the East Anglia region: Addenbrooke's Hospital, Cambridge; the Norfolk and Norwich Hospital, Norwich; the Ipswich Hospital, Ipswich and the West Suffolk Hospital, Bury St Edmunds.

The following variables, obtained from the East Anglia Cancer Registry data base, were treated as explanatory and/or confounding in our analysis: histological stage of tumour, grade of tumour, morphology of tumour, social class as determined by patient occupation and deprivation category as determined by postcode of residential area at the time of diagnosis. Stage was as defined in the TNM system. Where available, the grade of the tumour was coded as grade 1 (well differentiated), grade 2 (moderately differentiated) and grade 3 (poorly differentiated). Tumour morphology was coded in terms of the following six categories: ductal, medullary, lobular, mucinous/tubular, adenocarcinoma not otherwise specified and others.

Socioeconomic status was represented in terms of census area of residence and occupation. Social class was initially represented in terms of five categories, I, II, IIIN, IIIM, IV and V. Owing to the relatively large numbers of missing data on occupation, we had a limited number of cases of known social class. We therefore collapsed the five categories into two, with the first level representing categories I, II and IIIN, and the second level representing the categories IIIM, IV and V. This essentially splits the occupations into manual and nonmanual. We used as a second measure of socioeconomic status the Carstairs index based on indices of affluence/poverty in the area of residence (Morris and Carstairs, 1991). This was ascertained from the patient's postcode. We also adjusted for age at diagnosis.

Survival rates were estimated using the Kaplan–Meier product limit method. Cox's (1972) regression model for censored survival data was applied to our data to study the dependence of hazard of death on the explanatory variables, taking account of potential confounders. We had data on 6289 deaths out of a total of 10 865 breast cancer cases; of these, 1383 died from breast cancer as the underlying cause, this being our end point. We also performed a secondary survival analysis for death from any cause in breast cancer cases. Numbers available for different analyses vary due to different numbers with missing data on the explanatory variables, since for each analysis, we wished to use as much information as was available.

Our analysis consisted of two main phases. In the first phase, we performed a collection of analyses adjusted for age alone to estimate the hazard ratios (HR) associated with the explanatory variables of interest, without any adjustment for the effect of the remaining variables. In the second phase of the analysis, we used Cox's model to perform a multivariate regression analysis in which the joint impact of more than one explanatory variable on hazard of death is simultaneously modelled. This analysis led to estimation of an adjusted RR for each explanatory variable of interest. This was used to ascertain whether adjustment for stage or morphology accounted for more of the observed effect of socioeconomic status. We used the attributable proportion (Freedman *et al*, 1992) to estimate the extent to which adjustment for the tumour attributes accounted for differences in survival by socioeconomic group.

## RESULTS

Table 1

**Table 1:** Basic data and age-adjusted estimates of survival effects by univariate analysis

**Factors**	**Category**	**No. of cases**	**No. of deaths^a^**	**5-year survival probability**	**(95% CI)**	**HR**	**(95% CI)**
Stage	1	3469	260	0.96	0.95–0.97	1.00	—
	2	4230	651	0.91	0.89–0.92	2.40	2.07–2.78
	3	1504	237	0.89	0.81–0.87	3.56	2.97–4.26
	4	698	142	0.62	0.55–0.69	11.31	9.17–13.93
							
Grade	1	293	12	0.98	0.94–0.99	1.00	—
	2	665	103	0.89	0.85–0.91	4.08	2.24–7.42
	3	615	169	0.79	0.75–0.83	7.27	4.04–13.08
							
Morphology	Ductal	4454	605	0.90	0.89–0.92	1.00	—
	Medullary	154	15	0.93	0.86–0.97	0.63	0.37–1.05
	Lobular	1314	205	0.91	0.89–0.93	1.12	0.95–1.31
	Mucinous/tubular	298	15	0.99	0.96–1.00	0.30	0.18–0.52
	Adenocarcinoma	1716	186	0.95	0.93–0.97	0.67	0.56–0.79
	Others	2929	357	0.88	0.86–0.90	1.08	0.94–1.25
							
Social class	High	2988	448	0.90	0.88–0.91	1.00	—
	Low	1156	240	0.86	0.83–0.88	1.32	1.12–1.55
							
Deprivation	Most affluent	2529	320	0.91	0.89–0.92	1.00	—
	2	2983	376	0.91	0.90–0.93	1.01	0.87–1.18
	3	2788	357	0.91	0.89–0.93	1.02	0.87–1.19
	4	1904	240	0.91	0.89–0.93	1.00	0.84–1.18
	Most deprived	661	90	0.91	0.88–0.94	1.21	0.95–1.54

a Underlying cause.

shows numbers of cases and deaths from breast cancer as underlying cause, age-adjusted hazard ratio estimates and simple 5-year survival rates for the main explanatory variables. Increases in risk of death were noted for advanced stage, poor histological grade, the ductal, lobular and ‘others’ morphology categories and low socioeconomic status by either social class (occupational measure) or deprivation category (area of residence measure). Adjustment for histological grade has not been presented since, for many of the early cancer notifications, grade was not available. The unadjusted effect of social class on risk of death from breast cancer is clearly significant (*P*<0.001), whereas that of deprivation category is only suggestive (*P*=0.1). The difference between the highest and the lowest deprivation groups approaches significance (*P*=0.1).

Table 2

**Table 2:** Survival by deprivation category adjusted for age and stage (*N*=9891)

**Deprivation**	**HR**	**(95% CI)**
1 (most affluent)	1.00	—
2	1.04	0.89–1.22
3	1.01	0.86–1.19
4	0.98	0.82–1.17
5 (most deprived)	1.23	0.96–1.56

shows estimated hazard ratios and corresponding 95% confidence intervals (CIs) by deprivation category, adjusting for stage and age for breast cancer deaths only. The results show that adjusting for stage did not substantially change the estimated hazard ratio or the CI. Freedman's estimate of the percentage of the effect of the most deprived category attributable to stage is 0%. This suggests that the survival disadvantage of women in the ‘most deprived’ category cannot be explained in terms of a more advanced stage at diagnosis.

Table 3

**Table 3:** Survival by social class after adjusting for age and stage (*N*=3965)

**Social class**	**HR**	**(95% CI)**
High	1.00	—
Low	1.22	1.03–1.44

shows hazard ratios by social class, adjusted for age and stage, for breast cancer deaths. The hazard ratio is substantially attenuated by adjustment for age and stage. The Freedman estimate of the proportion of the social class effect attributable to age and stage was 28%. Thus, a proportion of the poorer survival in lower social class women can be explained by their more advanced stage at presentation. There still remains a significant effect of social class, however, after adjustment.

Table 4

**Table 4:** Survival by deprivation category adjusted for age and morphology (*N*=10843)

**Deprivation**	**HR**	**(95% CI)**
1 (most affluent)	—	—
2	1.02	0.87–1.19
3	1.03	0.88–1.20
4	1.02	0.85–1.21
5 (most deprived)	1.23	0.96–1.55

gives hazard ratio estimates by deprivation categories, adjusting for age and morphology, for all deaths and for breast cancer deaths only (underlying cause). Adjusting for ‘morphology’ did not substantially change the hazard ratio point and interval estimates, in particular for the most deprived category. This suggests that the apparent hazard disadvantage of women in the ‘most deprived’ category cannot be explained in terms of a more unfavourable morphology of the tumour at diagnosis. The Freedman measure of attributable proportion of the effect was none at all.

Table 5

**Table 5:** Survival by social class after adjusting for age and morphology (*N*=4141)

**Social class**	**HR**	**(95% CI)**
High	1.00	—
Low	1.31	1.12–1.54

shows the hazard ratio estimates for the social class categories adjusted for age and morphology; the adjustment for morphology made little difference. The Freedman attributable proportion was 3%.

It should be noted that for both social class and deprivation category, simultaneous adjustment for stage and morphology did not yield effects materially different from those observed after adjusting for stage alone. Adjusted for both, the hazard ratio for lower social class was 1.22 (95% CI 1.04–1.43), and for the most deprived category the hazard ratio was 1.23 (95% CI 0.97–1.57).

In the secondary analysis using deaths from all causes, the results were different. The age-adjusted hazard ratio for low social class was 1.20 (95% CI 1.09–1.32), and for the highest deprivation category it was 1.24 (95% CI 1.11–1.39). Stage accounted for 63% of the social class effect on all-cause deaths (HR=1.07, 95% CI 0.97–1.18) and for 31% of the deprivation category effect (HR=1.16, 95% CI 1.03–1.31). Adjustment for morphology produced only minor changes to the age-adjusted hazard ratios and did not attenuate at all those already adjusted for stage.

Table 6

**Table 6:** Social class and deprivation category by stage and morphology (percentages in parentheses)

		**Social class**		**Deprivation category**	
**Pathology factor**	**Category**	**I,II,IIINM**	**IIIM,IV,V**	**1–4**	**5**
Stage	1	1156 (40)	377 (34)	3268 (35)	201 (33)
	2	1257 (44)	468 (43)	3977 (43)	253 (42)
	3	302 (11)	163 (15)	1417 (15)	87 (15)
	4	157 (5)	87 (8)	636 (7)	62 (10)
	Total	2872	1095	9298	603
					
Morphological type	Ductal	1524 (51)	525 (45)	4207 (41)	247 (37)
	Medullary	52 (2)	21 (2)	145 (1)	9 (1)
	Lobular	411 (14)	136 (12)	1232 (12)	82 (13)
	Mucin/tub^a^	87 (3)	29 (3)	284 (3)	15 (2)
	Adenocarcinoma	400 (13)	165 (14)	1593 (16)	123 (19)
	Other	514 (17)	280 (24)	2744 (27)	185 (28)
	Total	2988	1156	10 204	661

a Mucinous or tubular carcinoma.

shows stage and morphological type cross-tabulated with socioeconomic status as measured by both social class and area-based deprivation category. Stage was significantly associated with social class (*P*<0.001) and deprivation category (*P*=0.03), with lower socioeconomic status being associated with more advanced stage. Morphology was significantly related to social class (*P*<0.001). This was partly due to fewer ductal carcinoma cases in lower social classes, but in the main due to a larger number of ‘other’ category cases in the lower social classes. There was no significant association between morphology and deprivation category (*P*=0.2), although again there were fewer ductal carcinoma cases in the lowest category.

## DISCUSSION

Our results add further to the evidence of an increased risk of death in breast cancer patients of low socioeconomic status, whether measured occupationally as social class or by an area-based method. They also suggest that this increased risk is not entirely attributable to morphological type or stage of disease. Although stage tends to account for more of the effect, particularly of the social class measure, there is still a substantial proportion of the effect unaccounted for by stage (or by stage and morphology together). This is consistent with the findings of Thomson *et al* (2001), who observed that oestrogen receptor status and treatment factors accounted for only 20% of the difference in survival between patients of high and low socioeconomic status (as measured by area of residence). Similarly, Newman *et al* (2002) found that the survival differences by ethnic group in the US could not be accounted for by stage of disease.

Nevertheless, we did find significantly poorer stage at presentation in patients of lower socioeconomic status, and this accounted for some of the differences in survival. This is in disagreement with the findings of Carnon *et al* (1994), who reported no significant differences between socioeconomic groups with respect to tumour size or node status. Our results are, however, partly consistent with those of Macleod *et al* (2000a) who reported a higher rate of clinically determined locally advanced disease in patients resident in deprived areas. Brewster *et al* (2001) reported no significant differences in stage among socioeconomic groups, but a reanalysis of their tabular data on tumour size in breast cancer patients shows a significant trend of increasing size with deprivation (*χ*=5.17, 1 degree of freedom, *P*=0.02). Thus, there is some support for our association of stage and socioeconomic status in the literature.

In our study, the distribution of morphological type was associated with socioeconomic status, but it is difficult to interpret this since 40% of the cases were classified simply as adenocarcinoma or ‘other’. An association of morphological type with socioeconomic status is not entirely unexpected, since Thomson *et al* (2001) found a significant association between deprivation category and oestrogen receptor status. More thorough morphological typing might account for more of the survival effect of socioeconomic status. Also, in recent years, histological grade has been registered in the majority of cases, so that when further follow-up of recent cases becomes available, we will be able to assess the extent to which histological grade may account for socioeconomic differences in survival. Since it is known that some risk factors, notably family history, vary in their effect on risk by grade (Duffy *et al*, 1999) and it is likely that in some cases, grade may actually deteriorate as the tumour grows (Tabar *et al*, 1996), there is at least a possibility that grade is implicated in the prognostic effect of socioeconomic status.

The association of survival with socioeconomic status (measured by either means) remained significant or close to significant after adjustment for stage or morphological type or both. It is notable that the adjustment accounted for a greater proportion of the survival effect for death from any cause than for breast cancer as the underlying cause of death. This may be due to misclassification of cause of death, a direct effect of tumour attributes on deaths from other causes or an increased risk of death from other causes in association with more aggressive treatment of more advanced tumours. Also, it is of interest that whereas deprivation category was not significant as a predictor of breast cancer death (although it was so for all-cause deaths), its effect was more robust to adjustment. Stage accounted for at least part of the effect of social class but for none of the effect of deprivation category, suggesting that the nonoccupational aspects of poverty captured by the deprivation score are associated with survival independently of stage of disease. This may be related to either host factors that affect survival generally or poorer access to prompt and appropriate therapy.

The conclusions of the above are:

1. Survival rates in East Anglia confirm the poorer survival of breast cancer patients of lower socioeconomic status.
2. This is only partly accounted for by differences in stage at presentation and morphological type.
3. Assessment of the extent to which histological grade accounts for the poorer survival of patients with lower socioeconomic status is a target for future research.
4. Research is needed on access to and timing of appropriate treatment in relation to socioeconomic status.

## References

[bib1] Brewster DH, Thomson CS, Hole DJ, Black RJ, Stoner PL, Gillis CR (2001) Relation between socioeconomic status and tumour stage in patients with breast, colorectal, ovarian, and lung cancer: results from four national, population based studies. Br Med J 322: 830–831 11290637 10.1136/bmj.322.7290.830 PMC30560

[bib2] Carnon AG, Ssemwogerere A, Lamont DW, Hole D, Mallon EA, George WD, Gillis RC (1994) Relation between socioeconomic deprivation and pathological prognostic factors in women with breast cancer. Br Med J 309: 1054–1057 7950739 10.1136/bmj.309.6961.1054 PMC2541541

[bib3] Cox DR (1972) Regression models and life tables. J Roy Statist Soc B 34: 187–220

[bib4] Duffy SW, Tabar L, Smith RA, Krusemo UB, Prevost TC, Chen HH (1999) Risk of breast cancer and risks with breast cancer: the relationship of histologic type with epidemiology, disease progression and survival. Semin Breast Dis 2: 292–300

[bib5] English WP, Cleveland KE, Barber WH (2002) There is no difference in survival between African-American and white women with breast cancer. Amer Surgeon 68: 594–597 12079146

[bib6] Freedman LS, Graubard BI, Schatzkin A (1992) Statistical validation of intermediate endpoints for chronic diseases. Stat Med 11: 167–178 1579756 10.1002/sim.4780110204

[bib7] Kogevinas M, Porta M (1997) Socioeconomic differences in cancer survival: a review of the evidence. IARC Sci Publ 138: 177–206 9353665

[bib8] Macleod U, Ross S, Gillis C, McConnachie A, Twelves C, Watt GC (2000a) Socio-economic deprivation and stage of disease at presentation in women with breast cancer. Ann Oncol 11: 105–107 10690397 10.1023/a:1008385321476

[bib9] Macleod U, Ross S, Twelves C, George WD, Gillis C, Watt GC (2000b) Primary and secondary management of women with early breast cancer from affluent and deprived areas: retrospective review of hospital and general practice records. Br Med J 320: 1442–1445 10827047 10.1136/bmj.320.7247.1442 PMC27387

[bib10] Meng L, Maskarinec G, Wilkens L (1997) Ethnic differences and factors related to breast cancer survival in Hawaii. Int J Epidemiol 26: 1151–1158 9447393 10.1093/ije/26.6.1151

[bib11] Morris R, Carstairs V (1991) Which deprivation? A comparison of selected deprivation indexes. J Publ Health Med 13: 318–326 1764290

[bib12] Newman LA, Bunner S, Carolin K, Bouwman D, Kosir MA, White M, Schwartz A (2002) Ethnicity related differences in the survival of young breast carcinoma patients. Cancer 95: 21–27 12115312 10.1002/cncr.10639

[bib14] Pollock AM, Vickers N (1997) Breast, lung and colorectal cancer incidence and survival in South Thames Region, 1987–1992: the effect of social deprivation. J Public Health Med 19: 288–294 9347452 10.1093/oxfordjournals.pubmed.a024632

[bib15] Tabar L, Fagerberg G, Chen HH, Duffy SW, Gad A (1996) Tumour development, histology and grade of breast cancers: prognosis and progression. Int J Cancer 66: 413–419 8635853 10.1002/(SICI)1097-0215(19960516)66:4<413::AID-IJC1>3.0.CO;2-Z

[bib16] Thomson CS, Hole DJ, Twelves CJ, Brewster DH, Black RJ (2001) Prognostic factors in women with breast cancer: distribution by socioeconomic status and effect on differences in survival. J Epidemiol Comm Health 55: 308–315 10.1136/jech.55.5.308 PMC1731899 11297648

[bib17] Twelves CJ, Thomson CS, Gould A, Dewar JA (1998) Variation in the survival of women with breast cancer in Scotland. Br J Cancer 78: 566–571 9744492 10.1038/bjc.1998.541 PMC2063068

